# Alterations in lipid metabolism and blood profile in gynecological cancers – potential strategies in diagnosis and treatment

**DOI:** 10.3389/fphys.2026.1741759

**Published:** 2026-02-16

**Authors:** Yelyzaveta Razghonova, Anna Abacjew-Chmylko, Monika Czapiewska, Dariusz Wydra, Julian Swierczynski, Adriana Mika, Tomasz Sledzinski

**Affiliations:** 1 Department of Pharmaceutical Biochemistry, Faculty of Pharmacy, Medical University of Gdansk, Gdansk, Poland; 2 Department of Gynecology, Obstetrics and Neonatology, Medical University of Gdansk, Gdansk, Poland; 3 Institue of Nursing and Medical Rescue, State University of Applied Sciences in Koszalin, Koszalin, Poland; 4 Department of Environmental Analytics, Faculty of Chemistry, University of Gdansk, Gdansk, Poland

**Keywords:** cervical cancer, endometrial cancer, fatty acids, lipogenic enzymes, ovarian cancer

## Abstract

Gynecological cancers (GCs), especially endometrial, cervical and ovarian cancers, represent a major health burden due to their increasing incidence and poor treatment outcomes, particularly in advanced stages. Numerous papers suggest that reprogramming of lipid metabolism plays an important role in the development and progression of GCs. In this review, we discuss the alterations in lipid metabolism, focusing on a) serum/plasma lipid profiles and changes in membrane lipid composition in GCs patients, b) dysregulation of fatty acid uptake and β-oxidation by GCs cells, and c) upregulation of lipogenic enzymes in cancer tissue of GCs patients and GCs cells lines. It appears that lipid alterations in the development and progression of GCs are very complex and cancer type specific. This is due to the complexity of a) the structure and properties of lipids, b) the variability between different human cancer types, and c) the need for a comprehensive set of clinical data. Moreover, the review highlights alterations of lipid metabolism as potential diagnostic and therapeutic strategies in the treatment of GC patients. Further studies are still needed to draw clear conclusions about the relationship between abnormalities in lipid metabolism and development of GCs and to bridge basic research and practice.

## 1 Introduction

In 2022, more than 1.5 million women worldwide were diagnosed with gynecological cancers (GCs) (Worldwide cancer data, 2024; Sung et al., 2021), and GCs accounted for 15.2% of all cancers diagnosed in women in that year (Worldwide cancer data, 2024). The most common GCs include malignancies of the endometrium, cervix and ovaries (Worldwide cancer data, 2024) and in this review we will focus primarily on the lipid metabolism of these cancers (Worldwide cancer data, 2024). Lipids not only constitute structural elements of membranes but also act as signaling mediators, energy reservoirs, and regulators of redox and stress responses. Cancer cells utilize at least five axes of lipid metabolism: (1) exogenous uptake mediated by lipid transporters, (2) *de novo* fatty acids (FA) synthesis, (3) cholesterol synthesis and esterification, (4) fatty acid β-oxidation, and (5) storage in lipid droplets. Rapidly proliferating cells, malignant or not, up/downregulate these programs; however, tumor selectivity arises from oncogenic activation and microenvironmental conditions (Hanahan and Weinberg, 2011). The presence of cancer-specific metabolites in serum, blood cells and urine (or other biological material) and/or changes in plasma (or serum) concentrations of already known metabolites, may serve as noninvasive potential biomarkers for improving the diagnosis of many diseases, including GCs. Such applications of lipid alteration are possible by using chromatography-based techniques, including gas chromatography-mass spectrometry (GC-MS) and liquid chromatography-mass spectrometry (LC-MS), have long been used to study cancer cell metabolism (Kałuzna-Czaplińska and Jóźwik, 2014) or to determine the carcinogenesis rate of cancer cells (Lubes and Goodarzi, 2018). However, study designs, analytical platforms, and patient metabolic heterogeneity vary widely across GC studies, which complicates cross-cohort comparability and translation into clinical practice.

Therapeutic approaches targeting metabolic processes such as lipogenesis, glycolysis, glutaminolysis, and oxidative phosphorylation have been tested in preclinical cancer models (Montaño-Samaniego et al., 2020; Sukjoi et al., 2021). These pathways are critical, but not specific to cancer cells. Therefore, the challenge is to develop therapies that selectively target tumors while preserving adjacent normal tissues, including stromal and immune cells in the tumor microenvironment. Progress has been modest so far, but inhibitors of lipid metabolic enzymes are advancing through preclinical development. Examples include FASN inhibitors such as TVB-2640 (denifanstat), which has entered early clinical trials (Mallick et al., 2023); SCD1 inhibitors (e.g., CAY10566, MF-438) that sensitize tumors to ferroptosis (Tesfay et al., 2019); ACLY inhibitors such as SB-204990 (Hatzivassiliou et al., 2005); and CPT1A inhibitors (etomoxir, perhexiline) that block fatty-acid oxidation (Ma et al., 2018). Recent reviews highlight these drug candidates as the most clinically relevant and mechanistically informative (Long et al., 2018; Sukjoi et al., 2021; Munir et al., 2022; Vasseur and Guillaumond, 2022; Mallick et al., 2023).

Despite significant advances in prevention, diagnosis and treatment, the cancer rates, including GCs, are still rising worldwide. Understanding of the relationships between disorders in lipid metabolism and GCs may help in the development of new potential diagnostic and therapeutic strategies. This review addresses a) changes in the serum and tissue lipid profile of GC patients, b) the molecular basis of the changes in lipid metabolism that occur during GC treatment, c) the potential diagnostics and therapeutic interventions based on changes in GC lipid metabolism.

## 2 Reprogramming of lipid metabolism in gynecological cancers

In general, tumor transformation of cells is a multi-step pathological process in which cells acquire new phenotypic properties, including unlimited proliferation potential, the ability to grow invasively, metastasize, undergo angiogenesis and evade immune attack, insensitivity to growth suppressors, genome instability and metabolic reprogramming (Hanahan and Weinberg, 2011). For rapid proliferation, cancer cells require a higher levels of ATP and building blocks, especially proteins and lipids (predominantly phospholipids), which are necessary for the formation of new cell membranes. In most cancer cells, membrane lipids originate mainly from lipogenesis (Kałuzna-Czaplińska and Jóźwik, 2014) and cholesterogenesis (Lubes and Goodarzi, 2018). Metabolic reprogramming in rapidly proliferating cancer cells is primarily characterized by a shift in energy supply from oxidative phosphorylation to anaerobic glycolysis (a state in which cancer cells rely on ATP production in anaerobic glycolysis even when they have access to oxygen) (Sukjoi et al., 2021) and reprogramming of lipid metabolism (Munir et al., 2019). It is noteworthy that lipid metabolism is highly adaptable process and facilitates cancer cells to cope with a difficult and changing microenvironment (Munir et al., 2019). These changes are due to the genetic program, which is mainly driven by a) growth factor receptor signaling, b) epigenetic changes, c) hormonal changes, and d) mutations (Hanahan and Weinberg, 2011). For a comprehensive understanding of the role of lipid metabolism in GCs, we present below an association between serum/plasma lipid altertions and tumorigenesis, cancer progression, and survival in GC patients.

### 2.1 Lipid profile in serum/plasma as a potential marker of risk for gynecological cancers

Serum lipid profile analysis is widely used in the prevention of cardiovascular disease, and increasing evidence suggests its value in cancer prognosis. Numerous studies have also shown significant associations between serum total cholesterol (TC), low-density lipoprotein cholesterol (LDL-C), high-density lipoprotein cholesterol (HDL-C) and triacylglycerol (TG) with incidence, progression, and survival in multiple cancers (Haddad et al., 2015; Que et al., 2015; Zhou et al., 2018; Johannesen et al., 2020; Liu et al., 2021). However, in GCs the evidence is largely observational and heterogeneous, and interpretation is complicated by differences in dietary status, timing of blood sampling (pre-diagnosis vs. peri-diagnosis vs. during treatment), and frequent confounding by obesity/adiposity, insulin resistance/diabetes, and lipid-lowering medication use (e.g., statins).

#### 2.1.1 Endometrial cancer (EC)

Patients suffering from EC have higher serum concentrations of TC, LDL-C and TG than control subjects (Lindemann et al., 2009; Melvin et al., 2012; Ghahremanfard et al., 2015). In contrast, a reduction in serum HDL-C levels was found in EC patients (Table 1). Because EC is strongly linked to obesity and hyperinsulinemia, dyslipidemia in EC cohorts may reflect host metabolic risk (and shared endocrine/inflammatory mechanisms) rather than tumor-specific lipid effects, especially when BMI and diabetes/insulin and lipid-lowering therapy are not rigorously controlled. In a large prospective cohort study, a positive correlation between serum TG levels and EC risk was observed, but no significant association was found between serum LDL-C and HDL-C with EC risk (Seth et al., 2012). Essentially similar results have been reported by other authors (Lindemann et al., 2009). Since high serum cholesterol levels promote chronic inflammation, estrogen imbalance (Yang and Wang, 2019; Lin et al., 2021), and TG and cholesterol esters serve as reservoir of lipids for cancer (Butler et al., 2020), these lipid patterns are mechanistically compatible with EC-promoting endocrine–inflammatory milieus; however, causality cannot be inferred from observational associations and reverse causation (tumor- or preclinical disease–driven lipid changes) remains possible. By contrast, one study indicated that patients over 55 years of age with low TC and LDL-C levels had a higher risk of EC (Swanson et al., 1994). Such directionally discordant findings underscore potential confounding by nutritional status, comorbidities, and medication use, and argue for longitudinal, well-phenotyped cohorts. One of the lipids strongly associated with EC is 27-Hydroxycholesterol (27-OHC). 27-OHC is formed in EC tissues and acts as an agonist for the Liver X receptor (LXR), promoting the proliferation of EC epithelial cell (Gibson et al., 2018). Notably, this evidence is primarily tissue-based and does not establish 27-OHC as a validated circulating biomarker for EC. Beyond cholesterol itself, lipoproteins such as HDL-C and LDL-C may also act as carriers of microRNAs and inflammatory mediators, influencing tumor–stroma interactions and systemic cancer biology (Vickers et al., 2011).

**TABLE 1:** Lipid profile in serum/plasma of patients with gynecological cancers.

Cancers	Triacylglycerol	Total cholesterol	Low-density lipoprotein cholesterol	High-density lipoprotein cholesterol	Number of patients	Clinical details	References
Endometrial	↑ risk with higher TG	↔ with EC risk	↔ with EC risk	↔ with EC risk	31473 women; 100 EC cases over 9 years follow-up	Prospective cohort; EC cases identified by linkage to Cancer Registry of Norway	Lindemann et al. (2009)
	↑ risk with higher TG	↑ risk with higher TC	↔ with EC risk	↔ with EC risk	225432 women; 1144 EC; mean follow-up ∼12 years	Registry-linked cohort built from CALAB lab tests (1985-1996) linked to Swedish registries	Seth et al. (2012)
	↑	↔	↔	↓	17 EC vs. 26 healthy controls	Endometrial carcinoma (EC) + type 2 diabetes (T2D) (case–control)	Qahremani et al. (2023)
	↑	↔	↔	↓	231 EC vs. 246 endometriosis	Hospital-based retrospective; EC vs. benign endometriosis group	Sun et al. (2016)
Cervical	↓	↓	↑	↓	30 cases vs. 30 healthy controls	patients with advanced squamous cervical cancer	Okonkwo et al. (2011)
	↑	↑	↑	↓	99 cases vs. 35 healthy controls	patients with carcinoma cervix	Raju et al. (2014)
	↑	↑	↑	↓	1589 cases vs. 1589 controls	patients with cervical cancer	Cheng et al. (2024)
	↑	↑	↑	↓	1713 cases vs. 10397 healthy controls	patients with cervical cancer	Jiang et al. (2022)
Ovarian	↓	↓	↓	↓	40 cases vs. 50 healthy controls	patients with ovarian cancer	Qadir and Malik (2008)
	↑	ND	ND	↓	573 EOC vs. 1146 healthy controls	patients with epithelial ovarian cancer (EOC)	Chen et al. (2017)
	↔	↓	↔	↓	1767 cases vs. 229 167 controls	the meta-analysis (PubMed, EMBASE and Cochrane Library – 12 studies); patients with OC were compared to women without OC	Onwuka et al. (2020)
	↔ risk of cancer	↔ risk of cancer	↔ risk of cancer	↔ risk of cancer	808 cases	Prospective cohort (Sweden, AMORIS); pre-diagnostic lipid measurements for patients with ovarian cancer	Melvin et al. (2012)
	↔	↓	↔	↓	1542 cases vs. 2195 controls	the meta-analysis (PubMed, EMBASE and Cochrane Library – 7 studies); patients with OC were compared to women without OC	Faur et al. (2022)

↓ - decreased; ↑ - increased; ↔ - no change; ND, not determined in cited papers.

#### 2.1.2 Cervical cancer (CC)

Similar to EC patients with CC have higher serum concentrations of TC, LDL-C and TG than control subjects (Lindemann et al., 2009; Melvin et al., 2012; Ghahremanfard et al., 2015), whereas reduced serum HDL-C levels (Table 1). In a retrospective study of early-stage CC patients, increased serum TC and TG levels were associated with worse overall survival (Lin et al., 2021; Jiang et al., 2022). In addition, serum TC and LDL-C levels in CC patients were positively associated with disease progression (Raju et al., 2014). It was suggested that maintaining physiological concentrations of TG and TC in serum may have an impact on overall survival of CC patients (Lin et al., 2021). By contrast, one study showed that fifty years old or older patients with CC who have an elevated serum TG level have a higher survival rate than patients with a lower serum TG level (Jiang et al., 2022). This association was not observed in patients younger than 50 years old (Jiang et al., 2022).

#### 2.1.3 Ovarian cancer (OC)

In OC patients, the available data on serum TC, LDL-C and TG are heterogeneous. Some data indicate increased, others decreased serum/plasma concentrations of these lipids (Table 1). However, serum HDL-C concentrations were lower in OC patients compared to controls, similar to EC and CC patients. Melvin et al. found that serum levels of TC, TG, ApoB/ApoA ratio and LDL-C/HDL-C ratio were higher in OC patients compared with the control group (Melvin et al., 2012). Some authors reported the association of high TG with reduced survival in patients with OC (Ghahremanfard et al., 2015). The lower HDL-C/LDL-C ratio was also associated with a more advanced stage of epithelial ovarian cancer (EOC) (according to the International Federation of Gynecology and Obstetrics classification - FІGО) (Lemieux et al., 2001), and a lower serum HDL-C/TC ratio in EOC was linked to a tendency toward chemoresistance (Lemieux et al., 2001). Importantly, OC cohorts are particularly heterogeneous (histology, stage, treatment exposure, inflammatory burden/ascites, and cachexia), which can drive “directionally discordant” lipid findings and increase the likelihood of reverse causation (tumor-driven lipid changes rather than lipid-driven tumor risk). Overexpression of the LDL receptor (which plays a key role in cholesterol transport into cells) in patients with EOC undergoing platinum therapy is associated with a poor prognosis (Huang et al., 2021). These findings suggest that the transport of cholesterol into cells and possibly its metabolism to oxysterols and/or estrogens may be involved in drug resistance in EOC (Huang et al., 2021).

Published data suggest that changes in serum lipid profile depend on the type of GCs, disease progression and treatment outcomes (Bel’skaya et al., 2019; Onwuka et al., 2020; Faur et al., 2022). The most repeatable changes in serum lipid profile observed in patients with GCs relate to decreased HDL-C. This finding is also consistent with obesity-associated dyslipidemia (increased TG and decreased HDL-C), which is a major risk factor for GCs (Reeves et al., 2007). Decreased serum HDL-C concentration accompanied by increased concentrations of TC, LDL-C and TG (Table 1) leads to an increase in TC/HDL-C, LDL-C/HDL-C and TG/HDL-C ratios, which are generally considered indicators of a higher risk of cardiovascular disease (Seth et al., 2012). It is also generally accepted that an elevated serum TG/HDL-C ratio indicates a higher risk of metabolic syndrome (Qahremani et al., 2023). However, these ratios should be interpreted cautiously in oncology because cachexia/nutritional status, systemic inflammation, and treatment-related lipid shifts can strongly influence circulating lipids and may confound prognostic associations. Recent studies highlight the prognostic potential of non-HDL cholesterol (non-HDL = TC - HDL-C), which may be more strongly associated with cancer risk than individual lipid parameters (Fang et al., 2024). It can therefore be concluded that patients with cardiovascular disease and/or metabolic syndrome are potentially more susceptible to GCs. Furthermore, it has been postulated that higher HDL-C has anti-inflammatory and antioxidant properties including promotion of cholesterol efflux and reverse cholesterol transport (Raju et al., 2014). Therefore, it can be hypothesized that a significant decrease in serum HDL-C could promote the development of chronic inflammation and oxidative stress in GC patients. The data presented so far suggest that oxidative stress, chronic inflammation and cancer are closely linked (Neganova et al., 2021). Recently published results based on a large and long-term study of multiple cancer types, including GCs, show that a decrease in serum HDL-C levels was associated with a higher risk of death (Yang and Wang, 2019). An association between serum LDL-C and metastasis has also been reported (Ghahremanfard et al., 2015; Sun et al., 2016). In addition, serum lipid profiles of patients with GCs showed higher TG and apolipoprotein A (apoA) levels, but lower HDL-C, LDL-C and TC levels compared to benign ovarian tumors, endometriosis and uterine leiomyomas (Sun et al., 2016).

Оverall, the associations betwen blood lipid profiles in GCs patients (similar to other human cancers) are very complex, cancer type specific and not fully elucidated. The results of several studies are contradictory, but preliminary conclusions can be drawn: Patients suffering from EC and CC have increased serum TC, TG and LDL-C concentrations and decreased serum HDL-C concentrations. OC patients are characterized by decreased serum and HDL-C levels, while the changes in serum TC, TG and LDL-C concentrations are inconsistent. Currently, however, serum lipids are not incorporated into routine GC diagnosis or treatment decisions. Moreover, longitudinal data suggest that systemic lipid levels can change during chemotherapy or radiotherapy, potentially serving as predictors of treatment response (Tian et al., 2019). However, it is not excluded that the availability of new, more sophisticated diagnostic techniques, including GC-MS, LC-MS, and electrospray ionization mass spectrometry (ESI-MS), will allow the inclusion of a more extensive serum lipid profile (e.g., including fatty acids (FAs) and/or specific complex lipids such as phospholipids, sphingolipds etc.) in the diagnosis and treatment efficacy of some GCs in the near future. Therefore, further studies are needed to clarify the changes in serum lipid profile in GC patients and the impact of abnormal blood lipids on the development and treatment of GC.

### 2.2 The alterations in fatty acid profile in serum and red blood cells of patients with gynecological cancers

FAs are essential components of cell membrane lipids and have a significant impact on membrane fluidity and numerous cellular processes, including energy production, signal transduction, and the regulation of gene expression (De Carvalho and Caramujo, 2018). Because these properties depend on the chain length and degree of unsaturation, even modest shifts in the balance of saturated (SFA), monounsaturated (MUFA), and polyunsaturated fatty acids (PUFA) can alter membrane biophysics, receptor signaling, and oxidative vulnerability, thereby contributing to metabolic reprogramming in cancer cells (Westheim et al., 2023).

#### 2.2.1 EC

In EC cohorts, lower circulating SFAs have been reported (Gaudet et al., 2012; Audet-Delage et al., 2018). The major n-6 PUFA linoleic acid (LA, 18:2) was decreased in serum in both a large population-based study and a smaller preoperative postmenopausal cohort (Gaudet et al., 2012; Audet-Delage et al., 2018).

#### 2.2.2 CC and OC

Analysis of FA in red blood cells (RBCs) revealed that the total amount of SFA and the DPA were higher in CC and OC patients compared to controls, while linoleic acid (LA, 18:2 n-6) and DHA were significantly lower (Bannikoppa et al., 2017). Because RBCs lipids represent FA status over ∼120 days, they provide a “long-term metabolic fingerprint” of the host, that can be potentially useful for longitudinal monitoring (Fenton et al., 2016). This is particularly important because RBC phospholipid PUFA composition correlates with systemic organ PUFA levels (Lai et al., 2025), making it a robust biomarker for whole-body lipid status and potentially a better indicator of chronic metabolic shifts during cancer progression or treatment. At the same time, RBC FA patterns remain sensitive to background diet, obesity/insulin resistance, and inflammatory state, so case-control differences should be interpreted with attention to match/adjust for these factors and to standardize sample handling. Several studies report significant alterations in the serum FA profile of patients with EOC. The level of esterified SFA (lauric acid – 12:0, palmitic acid – 16:0, stearic acid – 18:0) was higher in the serum of patients with EOC than in control subjects (Yin et al., 2016). In contrast, esterified n-6 PUFA (GLA - gamma-linolenic acid, γ18:3 n-6, and ARA–arachidonic acid, 20:4 n-6) and n-3 PUFA (EPA - eicosapentaenoic acid, 20:5 n-3; and DHA-docosahexaenoic acid, 22:6 n-3) were lower in EOC patients than in healthy controls (Yin et al., 2016). This pattern results in an increased SFA/PUFA ratio, which Yin et al. (Yin et al., 2016) proposed as a potential biomarker to diagnose patients with EОC. However, other studies have reported that 12:0 and 18:0 were higher in the serum of EOC patients compared to healthy controls, but myristic acid (14:0) and 16:0 were lower, which highlights a controversy likely due to patient heterogeneity, dietary intake, menopausal status, tumor stage, and different lipidomics platforms (Bannikoppa et al., 2017). Recent studies also demonstrate that FA profile of membrane phospholipids, not just total serum FA, is a major determinant of cancer cell phenotype. Elevated MUFA/SFA ratios, driven by increased stearoyl-CoA desaturase-1 (SCD1) activity, confer resistance to ferroptosis, whereas SCD1 inhibition reduces MUFA levels, increases PUFA incorporation, and restores susceptibility to lipid peroxidation and ferroptosis inducers (Varynskyi and Schick, 2024). This finding is clinically relevant because it connects FA desaturation indices (16:1/16:0, 18:1/18:0) with therapeutic vulnerabilities, suggesting they may serve as functional biomarkers of lipid metabolic reprogramming. The concentrations of monounsaturated FAs (MUFA) (palmitoleic acid, 16:1), n-6 PUFA (GLA, 20:2 n-6, and adrenic acid 22:4 n-6) and n-3 PUFA (DPA - docosapentaenoic acid, 22:5 n-3, ALA - α-linolenic acid, 18:3 n-3, EPA and DHA) in the serum of EOC patients were lower than in control subjects (Yin et al., 2016). Taking the data presented above together, it can be concluded that the ratio of some esterified SFA/esterified PUFA and the levels of some SFA, MUFA, n-3 and n-6 PUFA may be useful potential biomarkers to diagnose patients with EOC. Together, these findings support the idea that combined FA ratios (SFA/PUFA, MUFA/SFA) and desaturation indices (16:1/16:0, 18:1/18:0) may serve as integrated biomarkers of EOC lipid metabolism. Still, standardization of lipidomic methods, stratification by histological subtype (e.g., high-grade serous vs. clear-cell EOC), and adjustment for metabolic comorbidities are urgently needed before clinical implementation (Zhao et al., 2022). Moreover, metabolomic studies have demonstrated that serum FFA signatures can differentiate EOC from benign ovarian disease and were successfully used to build early-stage EOC diagnostic models (Katoh et al., 2023). Importantly, tumor SCD1 expression correlates with circulating monounsaturated FFA patterns, suggesting that systemic lipid alterations reflect tumor-intrinsic metabolic reprogramming (Katoh et al., 2023).

Table 2 presents available data on serum or red blood cells FA levels in patients with GC.

**TABLE 2:** Alterations in serum or red blood cells fatty acid levels in patients with gynecological cancers.

Cancers	Fatty acid type	Specific fatty acid	Increase/decrease compared to controls	Number of patients	Clinical details	References
Endometrial	Saturated Fatty Acids (SFA)	Myristic acid (14:0)	↓ (serum)	36 cases vs. 18 controls	Preoperative serum; postmenopausal women	Audet-Delage et al. (2018)
		Stearic acid (18:0)	↓ (serum)	250 cases vs. 250 controls	Population-based case-control; patients with endometrial cancer	Gaudet et al. (2012)
	n-6 Polyunsaturated Fatty Acids (n-6 PUFA)	Linoleic acid (LA, 18:2)	↓ (serum)	250 cases vs. 250 controls	Population-based case-control; patients with endometrial cancer	Gaudet et al. (2012)
			↓ (serum)	36 cases vs. 18 controls	Preoperative serum; postmenopausal women	Audet-Delage et al. (2018)
Cervical	Saturated Fatty Acids (SFA)	Palmitic acid (16:0)	↑ (serum)	50 cases vs. 40 healthy controls	Patients with cervical cancer	Katoh et al. (2024)
		Stearic acid (18:0)	↑ (serum)			
		Palmitic acid (16:0)	↑ (serum)			
	Monounsaturated Fatty Acids (MUFA)	Sapienic acid (16:1)	↑ (serum)	50 cases vs. 40 healthy controls	Patients with cervical cancer	Katoh et al. (2024)
		Palmitoleic acid (16:1)	↑ (serum)			
		Oleic acid (18:1)	↑ (serum)			
	n-3 Polyunsaturated Fatty Acids (n-3 PUFA)	α-linolenic acid (ALA, 18:3)	↑ (serum)	50 cases vs. 40 healthy controls	Patients with cervical cancer	Katoh et al. (2024)
		Docosapentaenoic acid (DPA, 22:5)	↑ (red blood cells)	31 cases vs. 56 control	Patients with cervical cancer	Bannikoppa et al. (2017)
			↓ (serum)	50 cases vs. 40 healthy controls	Patients with cervical cancer	Katoh et al. (2024)
	n-6 Polyunsaturated Fatty Acids (n-6 PUFA	Linoleic acid (LA, 18:2)	↓ (red blood cells)	31 cases vs. 56 control	Patients with cervical cancer	Bannikoppa et al. (2017)
			↑ (serum)	50 cases vs. 40 healthy controls	Patients with cervical cancer	Katoh et al. (2024)
		Dihomo-γ-linolenic acid (DGLA, 20:3)	↑ (serum)			
		Arachidonic acid (ARA, 20:4)	↑ (serum)			
Ovarian	Saturated Fatty Acids (SFA)	Lauric acid (12:0)	↑ (serum; EFA and FFA)	40 cases vs. 35 healthy controls	Patients with epithelial ovarian cancer	Yin et al. (2016)
			↓ (serum; FFA)			Yin et al. (2016)
		Palmitic acid (16:0)	↑ (serum; EFA)	40 cases vs. 35 healthy controls	Patients with epithelial ovarian cancer	Yin et al. (2016)
			↓ (serum; FFA)			
			↑ (red blood cells)	30 cases vs. 56 controls	Patients with ovarian cancer	Bannikoppa et al. (2017)
		Stearic acid (18:0)	↑ (serum; EFA and FFA)	40 cases vs. 35 healthy controls	Patients with epithelial ovarian cancer	Yin et al. (2016)
			↑ (red blood cells)	30 cases vs. 56 controls	Patients with ovarian cancer	Bannikoppa et al. (2017)
		Total SFA	↑ (red blood cells)	30 cases vs. 56 controls	Patients with ovarian cancer	Bannikoppa et al. (2017)
	Monounsaturated Fatty Acids (MUFA)	Palmitoleic acid (16:1)	↓ (serum; FFA	40 cases vs. 35 healthy controls	Patients with epithelial ovarian cancer	Yin et al. (2016)
	n-3 Polyunsaturated Fatty Acids (n-3 PUFA)	α-linolenic acid (ALA, 18:3)	↓ (serum; FFA)	40 cases vs. 35 healthy controls	Patients with epithelial ovarian cancer	Yin et al. (2016)
		Eicosapentaenoic acid (EPA, 20:5)	↓ (serum; EFA and FFA)			Yin et al. (2016)
		Docosapentaenoic acid (DPA, 22:5)	↓ (serum; EFA and FFA)			Yin et al. (2016)
			↑ (red blood cells)	30 cases vs. 56 controls	Patients with ovarian cancer	Bannikoppa et al. (2017)
		Docosahexaenoic acid (DHA, 22:6)	↓ (serum; EFA and FFA)	40 cases vs. 35 healthy controls	Patients with epithelial ovarian cancer	Yin et al. (2016)
	n-6 Polyunsaturated Fatty Acids (n-6 PUFA)	Linoleic acid (LA, 18:2)	↓ (red blood cells)	30 cases vs. 56 controls	Patients with ovarian cancer	Bannikoppa et al. (2017)
		Gamma-linolenic acid (GLA, γ18:3)	↓ (serum; EFA and FFA)	40 cases vs. 35 healthy controls	Patients with epithelial ovarian cancer	Yin et al. (2016)
		Eicosadienoic acid (EDA, 20:2)	↓ (serum; EFA and FFA)			Yin et al. (2016)
		Arachidonic acid (ARA, 20:4)	↓ (serum; EFA)			Yin et al. (2016)
		Adrenic acid (AdA, 22:4)	↓ (serum; FFA)			Yin et al. (2016)
		Docosapentaenoic acid (DPA, 22:5)	↓ (serum; EFA and FFA)			Yin et al. (2016)
		Total n-6 PUFA	↓ (red blood cells)	30 cases vs. 56 controls	Patients with ovarian cancer	Bannikoppa et al. (2017)
			↓ (serum; FFA)	40 cases vs. 35 healthy controls	Patients with epithelial ovarian cancer	Yin et al. (2016)
			↑ (serum; EFA)			Yin et al. (2016)

↓ - decreased; ↑ - increased, FFA, free fatty acid (FFA); EFA, esterified fatty acid.

Overall, circulating FA profile alterations in GCs are heterogeneous, partly contradictory, and influenced by multiple factors including tumor subtype, host metabolism, and methodology. While preliminary evidence supports the use of FA ratios and desaturation indices as potential biomarkers, their clinical translation requires (i) standardized lipidomics protocols, (ii) larger longitudinal studies stratified by tumor type, and (iii) correlation with treatment outcomes and immune-metabolic markers.

### 2.3 Potential procarcinogenic or anticarcinogenic effects of fatty acids in gynecological cancers

FA can exert dual and context-dependent roles in GC progression, acting as either tumor-promoting or tumor-suppressive signals depending on their saturation status, chain length, and downstream metabolic fate.

#### 2.3.1 EC

The effect of ARA and DHA on cell proliferation were investigated in several EC cell lines and in animal models (Tessier-Prigent et al., 1999; Zheng et al., 2014). Іt has been shown that DHA inhibits in a dose-dependent manner cell proliferation both *in vitro* and *in vivo* and promotes apoptosis by inhibiting mTOR signaling pathways (Zheng et al., 2014; Lubes and Goodarzi, 2018). In contrast, ARA stimulated cell proliferation both *in vitro* and *in vivo* (Butler et al., 2020). Moreover, treatment with DHA resulted in a rapid and dose-dependent suppression of phosphorylation of S6 (ribosomal protein - a key downstream component of the akt/mTOR signaling pathway that plays an important role in cancer cell survival) and phosphorylation of Akt (Zheng et al., 2014). Overall, these results suggest that ARA and DHA may play opposing regulatory roles in cell signaling pathways in EC (Zheng et al., 2014; Butler et al., 2020; West et al., 2020). These effects are consistent with reports showing that DHA can modulate membrane lipid rafts, enhance oxidative stress, and trigger ferroptosis-like cell death under certain conditions.

#### 2.3.2 CC

It has been shown that treatment of HeLa CC cells with 18:1 increased proliferative capacity, cell migration, and invasion ability (Yang et al., 2018). Because most CC evidence comes from HPV + cell lines, translation to patient tumors may depend on viral oncogene context, baseline metabolic state, and whether MUFA exposure reflects diet versus tumor-driven SCD1 activity. Іncreased MUFA content in the plasma membrane protects cancer cells from the cytoxic effects of SFA thus promoting cancer cell survival (Tesfay et al., 2019). In turn, GLA, EPA, ALA and DHA were found to accelerate the uptake of vincristine by HeLa CC cells and thus increase the therapeutic effect (Das et al., 1998). These “chemosensitization” findings are hypothesis-generating; they require confirmation in contemporary CC models and with clinically used regimens, because drug transport and membrane effects are highly assay- and dose-dependent.

#### 2.3.3 OC

It has been suggested that 18:1, the major MUFA in the human body, stimulates cell proliferation by activating Akt (also known as protein kinase B- PKB), whereas 16:0, the most abundant SFA, next to 18:0 in the human body, triggers apoptotic cell death and inhibits Akt pathway in several OC cell lines in a dose-dependent manner (Uddin et al., 2017). Blocking the FFA receptor 1 (FFAR1) in OC cell lines has been shown to reverse the proliferative effects of 18:1, while 16:0 inhibits pro-oncogenic signaling via the Akt pathway, highlighting the role of specific FAs in influencing cancer cell behavior (Uddin et al., 2017). In human ovarian granulosa tumor cells n-3 PUFA significantly reduces cancer cell viability and proliferation (Zheng et al., 2014; Yao et al., 2022). Moreover, the treatment by n-3 PUFAs increases the number of apoptotic cells (Yao et al., 2022). Another study has shown that DHA slows down cell division in human OC cells (West et al., 2020). The Hey OC cell line showed G2 arrest after treatment with DHA, while the ІGRОV-1 OC cell line showed a G1 arrest. Remarkably, G2 arrest in Hey cells was observed only at higher doses of DHA, which is often associated with increased apoptosis (West et al., 2020). GLA (n-6 PUFA) and EPA (n-3 PUFA) enhanced the cytotoxic effects of anticancer drugs such as vincristine, cisplatin, and doxorubicin (Das et al., 1998). Most evidence to date comes from *in vitro* systems and requires confirmation in clinically relevant models and human cohorts. In particular, “high/low” circulating FA levels are not equivalent to tumor membrane composition or intratumoral lipid signaling, and directionality may differ by histology (e.g., high-grade serous vs. clear-cell OC) and treatment exposure. Some major controversies remain. Some cohort studies have not confirmed a direct association between MUFA intake and GC risk, suggesting that endogenous MUFA synthesis via SCD1 may be a stronger driver of tumor progression than dietary intake (Currie et al., 2013; Röhrig and Schulze, 2016). PUFA subtype effects also appear context-dependent, as n-6 PUFAs can promote inflammation whereas n-3 PUFAs counteract it (Yang and Stockwell, 2016). Tumor heterogeneity, menopausal status, BMI, and metabolic comorbidities further complicate interpretation, emphasizing the need for stratified analyses (Yang and Wang, 2019; Lai et al., 2025). In conclusion, available evidence suggests that MUFA-rich membrane states (often linked to SCD1 activity) may support pro-survival signaling in GC models, whereas n-3 PUFA (e.g., DHA and EPA) more consistently show anti-proliferative and pro-apoptotic effects in experimental systems; however, circulating “high/low” serum levels should not be interpreted as causal without well-controlled prospective human studies. In addition, both n-3 and n-6 PUFA enhance the cytotoxic effects of some anticancer drugs and modulate the tumor microenvironment.

### 2.4 Dysregulation of fatty acids uptake by gynecological cancer cells

FAs uptake is a highly regulated and energetically costly process that allows cells to acquire essential lipids for energy production, membrane biosynthesis, and signaling. Unlike glucose, which diffuses readily through GLUT transporters, long-chain FAs require transporter-mediated entry and intracellular activation to acyl-CoA before metabolic utilization. This regulation becomes particularly relevant in cancer, where tumor cells exploit both circulating FAs and those released by stromal adipocytes as metabolic fuel, especially during metastatic spread and under nutrient stress (Schwenk et al., 2010; Currie et al., 2013). FAs are taken up by cells from the environment through FA transporters (FATPs) or plasma membrane FA binding proteins (FABPpm) and CD36 (Rysman et al., 2010; Knapp et al., 2012; Schneider et al., 2014; Zhang et al., 2018; Hauptmann et al., 2022). After import, long-chain FAs are “trapped” intracellularly by conversion to acyl-CoA (via long-chain acyl-CoA synthetases), which commits them to β-oxidation, phospholipid synthesis, or storage in triglycerides and thereby functionally couples uptake to downstream metabolic fate.

The PІ3K/Akt/mTОR is a key signaling pathway involved in the regulation of FA uptake (Acharya et al., 2023). Conversely, AMPK signaling pathway activation shifts cells toward catabolic program, it suppresses *de novo* lipogenesis while stimulate FA β-oxidation, thus lowering the intracellular lipid burden and driving compensatory uptake of extracellular FA (Samovski et al., 2015). Other factors that regulate the FA uptake by cells and FA β-oxidation are PPAR-γ and PPAR-α (Varga et al., 2011) and the MAPK/ERK pathway (Alexander et al., 2001). Dysregulation of these signaling pathways in cancer cells can result in increased FA uptake and altered lipid metabolism, which may contribute to the development and progression of cancer (Acharya et al., 2023). While AMPK/PPAR/MAPK regulation is described largely in the broader metabolic cancer biology literature, GC-specific evidence has been presented below.

#### 2.4.1 EC

Although FAs are an efficient energy source for some cancer cells, published data suggest metabolic shift favoring glucose over FAs utilization in EC (Knapp et al., 2012). For example, increased expression of glucose transporters (GLUT-1, GLUT-3, GLUT-4) and decreased expression of CD36, FABP4 and FATP-1 was found in EC (Knapp et al., 2012; Miki et al., 2022). Also, our recent study showed decreased CD36 gene expression in cancer tissue of EC patients (Razghonova et al., 2025). FABP4, an adipocyte-specific FA-binding protein, is secreted by adipocytes and can deliver FAs to neighboring tumor cells, thereby coupling the metabolic activity of the stroma to tumor growth (Tian et al., 2020). Interestingly, overexpression of FABP4 in EC has been linked to suppression of PI3K/Akt phosphorylation, resulting in reduced proliferation, migration, and invasion *in vitro* and suppression of tumor growth *in vivo* (Wu et al., 2021). Conversely, the addition of lipids to EC cell lines promoted an increase in CD36 levels, stimulates FA uptake, and cell proliferation (Miki et al., 2022). Moreover, CD36 protein levels in EC positively correlated with lymph node metastasis (Miki et al., 2022). Together, these findings support a model in which EC cells show reduced baseline FA import relative to glucose uptake, but lipid availability and microenvironmental inputs can still induce CD36-linked FA uptake and aggressive behavior in some settings.

#### 2.4.2 CC

Іnterestingly, expression of CD36 increases in human CC cell lines exposed to transforming growth factor-β (TGF-β), an epithelial-mesenchymal transition (EMT)-inducing factor (Sun and Zhao, 2022). Knockdown of CD36 in CC cell lines results in reduced cell migration, invasion, colony formation, and increased apoptosis, thus reducing EMT (Deng et al., 2019). Knockdown of FABP4 in CC cells resulted in decreased aggressiveness and increased expression of E-cadherin (protein associated with maintaining cell adhesion and preventing cell migration) and downregulated expression of N-cadherin and vimentin (marker of invasive cell phenotype), as well as p-Akt (involved in cell survival and proliferation) (Li et al., 2021). These data position CD36 and FABP4 as functional contributors to pro-migratory/EMT-linked phenotypes in several CC models, consistent with an FA uptake-supported invasive program.

#### 2.4.3 OC

In OC, metastatic tumor cells exhibit a unique dependency on adipocyte-derived lipids as a metabolic fuel. Adipocytes within the omental niche actively release FA, which are then transferred to OC cells in a process largely mediated by FABP4 (Nieman et al., 2011). This lipid crosstalk is not merely metabolic: it drives tumor proliferation, enhances invasion, and contributes to platinum resistance (Mukherjee et al., 2020). Genetic silencing or pharmacologic inhibition of FABP4 significantly reduces lipid transfer, suppresses tumor growth in xenograft models, and restores chemosensitivity, underscoring FABP4 as a promising therapeutic target (Nieman et al., 2011; Mukherjee et al., 2020). Furthermore, co-culture and *in vivo* studies demonstrate that adipocyte-OC interactions induce CD36 upregulation, while other transporters (FABPpm, FATP1 and FATP4) are unaffected. Upregulation of CD36 amplify FA uptake and lipid droplet accumulation, elevate ROS and pro-inflammatory cytokines, and activate *de novo* lipogenesis and cholesterol synthesis programs (Gharpure et al., 2018; Ladanyi et al., 2018). These results underscore FABP4 as a central node linking the adipocyte-rich microenvironment to tumor metabolic reprogramming and metastatic competence (Gharpure et al., 2018). The increase in FABP4 expression was also observed in cell lines representing high-grade serous OC (Mukherjee et al., 2020). Іnterestingly, knockdown of CD36 reduced adipocyte-induced FABP4 expression, whereas knockdown of FABP4 did not affect adipocyte-mediated CD36 expression (Sun and Zhao, 2022). Thus, these data suggest functional and/or regulatory relationship between CD36 and FABP4 in the context of adipocyte-induced changes in cancer cells (Mukherjee et al., 2020). CD36 inhibition also protects against adipocyte-driven EMT and stemness, which are associated with pro-metastatic effects, possibly mediated by FABP4 (Sun and Zhao, 2022). Primary ОC and ОC visceral metastases are characterized by upregulated CD36 expression (Wang et al., 2016). Collectively, in OC adipocyte-driven lipid supply, FABP4-mediated transfer, and CD36-linked uptake form a coherent metastatic metabolic axis (particularly in the omental niche).

Overall, decreased expression of CD36, FABP4 and FATP-1 was found in EC (Knapp et al., 2012; Razghonova et al., 2025). In contrast, upregulation of CD36 and FABP4 was observed in CC (Sun and Zhao, 2022) and OC (Wang and Li, 2019; Mukherjee et al., 2020; Eltayeb et al., 2022). This pattern suggests that EC may rely less consistently on exogenous FA transport than OC and CC in the cited datasets, although lipid uptake can still be induced under specific microenvironmental conditions and requires further validation across EC subtypes. By comparison, multiple preclinical and translational studies support a stronger dependence on adipocyte-linked FA uptake programs (FABP4/CD36) in OC and in several models of CC (Knapp et al., 2012).

### 2.5 Fatty acids as energy substrates and storage reservoirs in gynecological cancers

Although cancer cells primarily use glucose as an energy source, which is metabolized in anaerobic glycolysis, even in the presence of oxygen - a process known as the Warburg effect, GC cells are not metabolically uniform, and FA are also an important energy substrate for them (Biga et al., 2019).

#### 2.5.1 EC

In co-culture model, adipocytes displayed elevated lipolysis and released FFAs, which are then taken up by EC cells and oxidized, thereby enhancing their proliferative and invasive capacity (Zhou et al., 2024). Notably, adipocyte-EC cells co-cultures also upregulated SIRT1 signaling in the cancer cells, linking fatty acid β-oxidation (FAO) to transcriptional control of stress responses and EMT (Savardekar et al., 2024). In EC, omics-based studies identified lipid droplet-associated gene signatures and cholesterol synthesis enzymes localized to LDs as potential biomarkers and drivers of tumor progression (Ayyagari et al., 2025).

#### 2.5.2 OC

Increased expression of the gene encoding carnitine palmitoyltransferase 1 (CPT1), an enzyme that plays a key role in the entry of acyl-CoA into the mitochondria for FAO, has been demonstrated in OC cell lines (Shao et al., 2016). Inactivation of CPT1 led to reduction of intracellular ATP levels, induced cell cycle arrest at the G0/G1 phase, and increased susceptibility to metabolic stress, thereby underscoring the essential role of FAO in sustaining OC bioenergetics and survival (Shao et al., 2016). OC cells were also shown to have higher reliance on FAs metabolism compared to control cells (Grasmann et al., 2021; Tondo-Steele and McLean, 2022). Beyond intrinsic tumor metabolism, the tumor microenvironment strongly reinforces FAO dependency. In co-culture systems, adipocyte - OC interactions facilitate direct lipid transfer from adipocytes to cancer cells, primarily via FABP4-mediated shuttling, resulting in enhanced proliferation and metastatic potential (Nieman et al., 2011; Ladanyi et al., 2018). Lipid shuttling is coupled to increased CD36 expression, lipid droplet accumulation, and ROS generation, which further activate stress-adaptive pathways such as NF-κB and promote chemoresistance (Ladanyi et al., 2018). Importantly, FA utilization in GCs is not restricted to immediate oxidation. Cancer cells actively sequester excess FA into lipid droplets (LDs), which function both as energy reservoirs and as buffers protecting against lipotoxicity and ferroptosis (Jin et al., 2023; Safi et al., 2024). During metabolic stress or nutrient deprivation, stored triglycerides in LDs are mobilized through adipose triglyceride lipase (ATGL) and hormone-sensitive lipase (HSL), supplying substrates for FAO and sustaining mitochondrial ATP production (Safi et al., 2024; Deswal et al., 2025). This LD-FAO axis is increasingly recognized as a survival mechanism in high-grade serous OC, where elevated LD accumulation independently predicts poor prognosis (Iwahashi et al., 2021).

In summary, FAO and LD metabolism together form a coupled survival network in GCs, integrating energy production, redox homeostasis, and therapy resistance. Future studies should address how FAO inhibitors (e.g., CPT1 blockers) or LD-targeting strategies (e.g., ATGL modulators) can be combined with standard chemotherapy to selectively disrupt this metabolic axis.

### 2.6 Changes in the composition of membrane lipids in gynecological cancers

Phosphatidylcholine (PC), phosphatidylethanolamine (PE), phosphatidylinositol (PІ), phosphatidylserine (PS) phosphatidylglycerol (PG), lysophosphatidylcholine (LPC) and various sphingolipids including ceramides are representatives of polar lipids that are important structural components of cell membranes. In addition, polar lipids are involved in numerous cellular processes, including signal transduction and regulation of cell membrane fluidity (Spector and Yorek, 1985; Welti and Wang, 2004; Preetha et al., 2005).

#### 2.6.1 EC

The level of lysophosphatidic acid (LPA), a bioactive lipid formed by phospholipases from phosphatidic acid (PA) according to the reaction PA + H_2_O → LPA + FA (Geraldo et al., 2021), is significantly increased in the plasma of women suffering from EC (Wang et al., 2010), CC (Wang X. et al., 2022) and OC (Xu et al., 1998). Several findings emphasize the important role of LPA in the proliferation and invasion of EC cells (Wang et al., 2010). Moreover, LPA plays a crucial role in shaping the microenvironment of EC by stimulating the secretion of urokinase plasminogen activator and matrix metalloproteinase-7, which are involved in cancer progression (Wang et al., 2010; Geraldo et al., 2021). Platelet-activating factor (PAF) is produced from polar lipids, especially PC and PE, and regulates inflammation, immune response, and cancer progression (Ashraf and Nookala, 2023). Іt was found that PAF stimulates the proliferation and migration of cancer cells and promotes angiogenesis and tumor invasion in EC (Baldi et al., 1994) and OC (Deuster et al., 2021). Sfingolipids, including ceramides, sfingosine, sphinganine, sphingomyelins, gangliosides and sphingosine-1-phosphate (S1P), which are also components of cell membranes, have numerous functions, including cell signaling, apoptosis and regulation of other physiological and pathophysiological processes (Takai et al., 2005). The concentrations of sphinganine, dihydroceramide, ceramide, sphingosine and S1P were significantly higher in EC tissue than in normal endometrium (Knapp et al., 2017). Ceramide promotes apoptosis, while S1P promotes tumor survival and angiogenesis (Knapp et al., 2019). Thus, ceramide and S1P has been implicated in EC progression (Santin et al., 2004). Interestingly, C2 ceramide (a synthetic ceramide analog) altered the cell cycle by reducing the proportion of cells in S phase and increasing proportion of cells in G0/G1 and/or G2/M phase in Ishikawa EC cells (Takai et al., 2005). C2 ceramide also induces apoptosis and alters the expression of genes encoding proteins associated with cell growth and malignancy (Yu et al., 2011). This suggests that C2 ceramide may be a promising agent for the effective treatment of EC (Takai et al., 2005).

#### 2.6.2 CC

Aberrant levels of PC, PE, PІ, PG and PS in cell membranes promote CC cell proliferation and migration by activating the PІ3K/Akt and MAPK/ERK pathways (Koundouros and Poulogiannis, 2019). LPA was found to a) reduce cell death and chromatin aggregation in cells treated with doxorubicin (DOX) (Wang Q. et al., 2022; Wang X. et al., 2022) downregulate the expression of gene encoding caspase-3 in DOX-treated CC cells (Wang X. et al., 2022) prevent apoptosis in DOX-treated CC cells and d) reduce DOX-induced intracellular ROS levels (Wang Q. et al., 2022). These results suggest that LPA can protect CC cells from DOX-induced apoptosis (Wang K. H. et al., 2022). C8 ceramide (a synthetic ceramide analog) has an antiproliferative and cytotoxic effect on human CC cells infected with papillomaviruses (Bi et al., 2019). C8 ceramide significantly reduces the number of tumor cells and induces necrotic changes (Bi et al., 2019).

#### 2.6.3 OC

Some findings suggested that the levels of specific lipid molecules in cell membranes could be potential diagnostic biomarkers in GC (Preetha et al., 2005; Yagi et al., 2020). For example, Yagi et al. (2020) reported that a) the ratio of LPC(20:4)/LPC(18:0) may be useful for distinguishing patients with benign ovarian masses from healthy controls; b) the ratio of SM(d18:1/24:1)/SM(d18:1/22:0) (the letter ‘d’ refers to the 2 (di-) hydroxyl groups in sphingosine) may help to distinguish OC patients from other groups of gynecological patients; and c) the ratio of PC (18:0/20:4)/PC(18:0/18:1) may be useful to distinguish OC patients from controls. In addition, LPA promotes tumor cell proliferation, migration and invasion by activating LPAR1 and LPAR3 (G protein-coupled receptors) in OC (Balijepalli et al., 2021). Diacylglycerol and Ca^2+^ (known as second messengers) are also involved in LPA signaling and activate downstream pathways including PІ3K/Akt and MAPK/ERK pathways (Kouba et al., 2019). It should be noted that early stage OC patients were more accurately diagnosed by the determination of LPA, PE and LPC in serum than those diagnosed based on CA125 alone (Shan et al., 2012; Yagi et al., 2019). Sutphen et al. (2004) reported that the ratio of LPA/LPI (LPI - lysophosphatidylinositol) in plasma determined by electrospray ionization mass spectrometry (ESI-MS) was higher in patients with EОC. Moreover, reduced plasma LPC levels in EОC patients distinguished them from patients with borderline ovarian tumors (BOTs) (Zhang et al., 2012). Detection of LPA in vaginal secretions is a promising non-invasive diagnostic biomarker for the identification of endometrioid and ovarian malignancies in postmenopausal women (Minis et al., 2019). LPA in serum/plasma has been shown to be a prognostic biomarker for OC (Xu et al., 1998; Xu, 2019; Ikeda et al., 2024) and also a potential biomarker for EC (Wang et al., 2010) and CC (Wang Q. et al., 2022), but evidence for clinical utility remains limited by small cohorts and variable analytical platforms; independent validation is still required. Plasma levels of natural ceramides (C16:0-Cer, C18:1-Cer and C18:0-Cer) were higher in women with advanced OC than in control subjects. In addition, the levels of some natural ceramides (C16:0-Cer, C18:1-Cer, C18:0-Cer, C24:1-Cer and C24:0-Cer) and S1P were elevated in ovarian tissue of women with advanced OC compared to healthy controls (Knapp et al., 2017). Gangliosides are glycosphingolipids, that contain a sphingoid base (mainly sfingosine) and a carbohydrate (mainly glucose or galactose). Elevated levels of gangliosides haven been found in ascitic fluid and plasma of patients with OC (Santin et al., 2004). Іncreased serum levels of gangliosides in OC may be due to the release of gangliosides from the surface of tumor cells (Santin et al., 2004). The glycosphingolipid composition on the surface of OC cells may be an important contributor to EMT status (Santin et al., 2004).

Overall, polar lipids play a complex role in cancer progression by influencing cellular signaling, membrane composition, and interactions with the tumor microenvironment. LPA, ceramides and gangliosides are also promising diagnostic and prognostic biomarkers. However, most studies remain exploratory (often in small case-control cohorts), and translation to clinically actionable biomarkers will require standardized sampling/analytics and independent validation in prospective GC cohorts. Polar lipids involvement in key signaling pathways, including PI3K/Akt and MAPK/ERK, underscores their potential as therapeutic targets. Future research should further investigate the diagnostic utility of polar lipid profiles and their potential for the development of targeted therapies for GC.

## 3 Up-regulation of genes encoding enzymes involved in lipid synthesis in gynecological cancers

As mentioned above, tumors must efficiently generate energy and building bloks to expand and spread, so they are characterized by elevated expression of genes encoding lipogenic enzymes (Tennant et al., 2010). The increase in the rate of FAs synthesis during tumor growth is due to oncogenic mutations, especially those that activate the PІ3K/Akt/mTОR signaling pathway (Samuels and Ericson, 2006; Khan et al., 2013). The PІ3K/Akt/mTОR signaling pathway stimulates the expression of genes encoding lipogenic enzymes via the action of the transcription factor called sterol regulatory element binding protein - 1 (SREBP-1), the master regulator of lipogenesis (Krycer et al., 2010; Biga et al., 2019). This is particularly relevant for EC, where disruption of the PI3K pathway (including frequent PTEN loss and PIK3CA/PIK3R1 alterations) is a dominant genomic feature and is mechanistically consistent with a shift toward *de novo* lipogenesis and membrane phospholipid remodelling (Velasco et al., 2006; Getz et al., 2013; Cheung et al., 2014). In OC, a well-supported example of mutation-linked lipid rewiring involves TP53, the hallmark alteration of high-grade serous ovarian carcinoma (Bell et al., 2011). Mechanistically, wild-type p53 restrains the mevalonate (MVA) pathway, whereas mutant p53 can promote MVA gene expression, in part through SREBP-dependent transcriptional control, thereby increasing intracellular sterol/isoprenoid availability with downstream effects on membrane properties and prenylation-dependent signalling (Moon et al., 2018). Consistent with this biology in ovarian models, mevalonate-pathway antagonists (statins) suppress precursor lesions and/or OC growth *in vivo*, supporting genotype-associated metabolic vulnerabilities as therapeutically relevant (Kobayashi et al., 2015). In CC, HPV oncogene activity intersects with host growth pathways: E6/E7-associated activation of PI3K/AKT/mTOR signaling is widely reported in HPV-driven malignancy and is compatible with SREBP-linked lipid metabolism reprogramming observed in CC models (Bossler et al., 2019). Taken together, available evidence supports the view that tumor genomic alterations shape intracellular lipid phenotypes largely through shared signaling hubs (PI3K/Akt/mTOR-SREBP; p53-mevalonate), whereas clinically robust, associations of mutations with lipid metabolism alterations in GC remain incompletely established due to limited cohort sizes and inconsistent integration of sequencing with standardized lipidomics. In GC, this pathway is frequently dysregulated: PTEN loss and PIK3CA mutations in EC and OC drive constitutive PI3K/Akt activation (Kinross et al., 2012; Guo et al., 2014). In CC, HPV E6/E7 oncoproteins activate PI3K/Akt signaling, further enhancing SREBP1-driven lipogenesis and linking viral oncogenesis to metabolic reprogramming (Gore et al., 2025). It should also be noted that the PІ3K/Akt/mTОR signaling pathway activates the expression of genes encoding glucose transporters that supply glucose, the main precursor of acetyl-CoA, which is a substrate for lipogenesis (Krycer et al., 2010).

**FIGURE 1:**
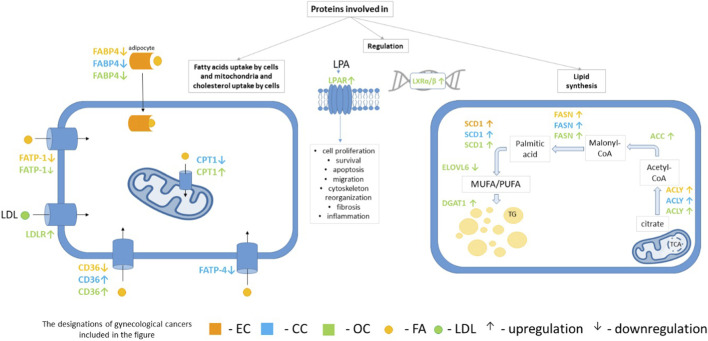
Schematic representation of the changes in the expression of various genes encoding enzymes and other proteins related to lipid metabolism, transport and regulation in gynecological cancers. Note: The schematic illustrates changes in the expression of key genes and proteins involved in fatty acid uptake, mitochondrial β-oxidation, lipid synthesis, and signaling in endometrial cancer (EC, orange), cervical cancer (CC, blue), and ovarian cancer (OC, green). CD36, FABP4, FATP1/4, and LDLR regulate lipid uptake, with CD36 and FABP4 upregulated in OC and CC but reduced in EC. CPT1 supports mitochondrial fatty acid oxidation, particularly in OC. Enzymes of *de novo* lipogenesis, including ACLY, ACC, FASN, and SCD1, are consistently upregulated, driving palmitate synthesis, desaturation, and membrane remodeling. ELOVL6 and DGAT1 contribute to FA elongation and lipid droplet formation. LPA and its receptors (LPAR1/3) activate PI3K/Akt and MAPK/ERK pathways, promoting tumor proliferation, invasion, and chemoresistance. Arrows indicate upregulation (↑) or downregulation (↓) of gene expression/protein activity in specific gynecological cancers. *Abbreviations: ACC–acetyl-CoA carboxylase; ACLY–ATP-citrate Lyase; CD36 – cluster of differentiation 36; CPT1 – carnitine palmitoyltransferase 1); DGAT1 – diacylglycerol O-acyltransferase 1; ELОVL6 – elongation of very long-chain fatty acids protein 6; FABP4 – fatty acid-binding protein-4; FASN–fatty acid synthase; FATP-1 – fatty acid transport protein-1; FATP-4 – sfatty acid transport protein-4; LDLR–low-density lipoprotein receptor; LPAR–lysophosphatidic acid receptor; LXRα/β–liver X receptor alpha/β; SCD1 – stearoyl-CoA desaturase 1. Up arrow–increase of gene expression; down arrow–decrease of gene expression.*

ATP citrate lyase (ACLY) is an important enzyme of lipogenesis that provides cytosolic acetyl-CoA as a substrate for 16:0 and other FAs synthesis as well as cholesterol biosynthesis (Koundouros and Poulogiannis, 2019). The expression of ACLY was higher in malignant tissues than in normal ovarian tissues and was associated with the degree of cancer and FІGО stage (Wang et al., 2012). Acetyl-CoA carboxylase (ACC, more precisely ACC1 also called ACCα) is a rate-limiting lipogenic enzyme, that converts acetyl-CoA to malonyl-CoA (Hunkeler et al., 2018). ACC1 is a highly regulated enzyme at the transcriptional and posttranslational level (Hunkeler et al., 2018; Koundouros and Poulogiannis, 2019). Posttranslationally, ACC1 is mainly regulated by phosphorylation/dephosphorylation, allosteric regulation and protein-protein interaction (Hunkeler et al., 2018). Interestingly, the tumor suppressor BRCA1 interacts with the phosphorylated (inactive) form of ACC1 (Brunet et al., 2008). The BRCA1/ACC1 complex (phosphorylated form) prevents the dephosphorylation of ACC1 and thereby inhibit ACC1 activity (Brunet et al., 2008). These results suggest that the ability of BRCA1 to stabilize the inactive form of ACC1 may be associated with suppression of the malignant phenotype. Overexpression of ACC1 has been found in many cancers, including GCs (Byrne et al., 2014; Li and Sui, 2021; Zhang et al., 2021; Zhen and Pan, 2023; Razghonova et al., 2025), where it is essential for cancer cell survival. Finally, it should be noted that malonyl-CoA is an allosteric inhibitor of CPT-1, which plays a key role in regulating β-oxidation of FAs and energy production in mitochondria (Wang Y. et al., 2022). Inhibition of ACC leads to a decrease in malonyl-CoA levels, thereby inhibiting lipogenesis and stimulating β-oxidation of FAs. Thus, inhibition of ACC activity as a therapeutic target is distinctly different from inhibition of FASN activity. Inhibition of FASN solely inhibits the biosynthesis of FAs. FASN, catalyzes the conversion of malonyl-CoA and acetyl-CoA into 16:0 (Koundouros and Poulogiannis, 2019). 16:0 is a substrate for palmitoylation of proteins, many of which are required for active cell proliferation (Sanders et al., 2015; Ko and Dixon, 2018). It is noteworthy that enzymes involved in palmitoylation of proteins are present in EC, OC and CC and may be related to carcinogenesis (Ko and Dixon, 2018). It is likely that palmitoylation of proteins also occurs with dietary palmitate as a substrate, which could have an impact on carcinogenesis. Synthesized 16:0 can be converted to palmitoyl-CoA and further to longer FAs by FA elongases (ELOVLs), and/or be the substrate for desaturation catalyzed by SCD1. Several studies indicate that most tumor cells synthesize about 95% of SFA and MUFA *de novo*, despite adequate dietary lipid intake (Menendez et al., 2005). This suggests that an increased rate of FA synthesis is required for tumorigenesis. As mentioned above, numerous studies have shown that the expression and activity of enzymes involved in lipogenesis are increased in various cancers, including GCs (Munir et al., 2019). Expression and activity of FASN and SCD1 are strongly elevated in OC, leading to an increase in saturated and monounsaturated FAs (Wang et al., 2001; Ji et al., 2020). Our study in EC also revealed increased FASN in tumor tissue, wherease 16:0 level was decreased or not changed depending on the stage of disease (Razghonova et al., 2025). In turn there was a trend to increased levels of 16:1 and 18:1, that was consistent with highly elevated expression of SCD1 in EC tissue (Razghonova et al., 2025). Thus, we can suppose that 16:0 is efficiently converted into MUFA in EC. An imbalance between saturated and unsaturated FAs can lead to lipid peroxidation and oxidative stress in the endoplasmic reticulum (ER) (Ji et al., 2020). Additionally, it should be noted that an enzyme-linked immunosorbent assay (ELISA) has been developed for the detection of FASN protein in human serum. This method is sensitive, accurate and precise for the quantitative determination of FASN protein levels in human serum. Using this method, it was shown that the FASN protein levels in serum were significantly higher (approximately 2-3 fold) in patients with some cancers, including OC, compared to control subjects (Wang et al., 2001). SCD1 and other desaturases, namely, D5D and D6D which are involved in PUFA conversions, encoded by the FADS1 and FADS2 genes, respectively, play a crucial role in controlling the levels of unsaturated FAs (Bannikoppa et al., 2017). In CC and ОC, global D6D activity estimated from serum product/substrate concentrations was increased (Bannikoppa et al., 2017). By contrast the pattern of changes of the expression of D5D and D6D in EC tissue was ambiguous and dependent on the stage of the disease (Razghonova et al., 2025). To date, the role of ELОVLs and FA elongation in GCs has not been intensively studied. In general, ELОVL1, -3, -4 and -6 are responsible for the elongation of SFA and MUFA, while ELОVL-2 and -5 mainly contribute to the synthesis of very long chain PUFA (Gao et al., 2021). ELОVL6 expression was lower in high-grade serous OC compared to normal ovarian tissue (Li et al., 2016). These results suggest that low ELОVL6 expression is associated with poor differentiation and drug resistance in high-grade serous OC (Li et al., 2016). Our study in EC tissue showed elevated ELOVL1 gene expression and elevated 24:0 level (Razghonova et al., 2025). The changes in the expression of genes encoding various enzymes and other proteins related to lipid metabolism, transport and regulation in GCs are summarized in Figure 1.

In summary, the observed similarities in lipogenic enzyme gene expression across GC types, particularly the consistent upregulation of FASN, ACLY and SCD1, underscore the universal metabolic reprogramming required for rapid tumor growth and proliferation. However, differences between different GCs, such as the differential regulation of CD36 and ELOVL6, also highlight the diverse metabolic adaptations. The consistent overexpression of some enzymes positions them as potential universal biomarkers for malignancy, while cancer-specific variations offer the opportunity to tailor therapies to individual tumor profiles. This interplay between common metabolic traits and cancer-specific adaptations underscores the complexity of cancer metabolism and highlight the need for personalized approaches to cancer diagnosis and treatment.

## 4 Potential therapeutic strategies targeting lipid metabolism in gynecological cancers

Current systemic treatments for GCs remain limited by recurrence, chemoresistance, and inter-patient metabolic heterogeneity; therefore, lipid metabolic dependencies are being explored as adjunct or context-specific therapeutic vulnerabilities rather than universal solutions. Since FAs are essential for cancer progression, the enzymes of lipid metabolism and lipid transporters may be a therapeutic targets. Lipid metabolism can be inhibitited by the following means (for details see Supplementary Table S1) a) FAs synthesis (e.g., by inhibiting FASN, ACC, ACLY and SCD1 activity; b) FAs β-oxidation (by decreasing CPT1 activity); c) FAs and cholesterol transport into cells (by inhibiting CD36, FABP4, LDLR); and d) targeting FAs storage (DGAT1, ACAT) (Fu et al., 2021). Importantly, most of these targets remain at the preclinical stage in GCs, and “druggability” differs markedly across nodes (enzymes vs. transporters vs. microenvironmental lipid transfer), necessitating careful separation of mechanistic promise from clinical readiness.

### 4.1 Preclinical evidence

In preclinical GC models, suppression of lipogenesis (reactions catalyzed by ACLY, ACC1 and FASN) and FA desaturation (via SCD1) reduces proliferation and invasion and can increase susceptibility to oxidative stress and lipid peroxidation-driven cell death (including ferroptosis-like phenotypes). Inhibition of ACLY activity in OC can lead to an increase in citrate levels and a decrease in acetyl-CoA and OAA levels (precursors for the biosynthesis of membrane lipids and nucleic acids) (Granchi, 2018). All these events can lead to OC cell death and inhibit tumor progression. The slowing of lipid metabolism, and thus cancer progression can be achieved by using single inhibitors, e.g., inhibitors of ACC1, or inhibitor combinations (e.g., inhibitors of ACLY and ACC1) which could pave the way for effective GCs therapy (Supplementary Table S1). Furthermore, by blocking the formation of lipid droplets, one can reduce the activation of survival-related kinases such as Akt and ERK1/2 associated with malignancy development (Iwahashi et al., 2021). This suggests that targeting lipid storage pathways may be another strategy for treating GCs (Huang et al., 2016; Iwahashi et al., 2021). In general, it can be said that slowing down lipogenic enzyme activity in GCs cells leads to a reduction in cancer cell proliferation, induction of apoptosis, reduction in cell migration and invasion, and affects other processes (Supplementary Table S1). Therefore, the results of studies with inhibitors of enzymes related to lipid metabolism seem promising as a potential strategy for the therapy of GCs.

Early clinical translation exists for core lipogenic targets, particularly FASN. In a phase I study of the oral FASN inhibitor TVB-2640 (denifanstat), treatment was generally feasible with manageable on-target toxicities, and the dose-expansion cohort included OC patients (n = 12). In this ovarian subgroup, CA-125 decreases were reported in 5/12 patients (58%–98%), and a confirmed Response Evaluation Criteria in Solid Tumors (RECIST) partial response was observed in one patient with primary peritoneal carcinoma (Dean et al., 2016). Accordingly, the correct framing is that GC-dedicated, randomized interventional trials of lipid-metabolism inhibitors remain limited, while the most direct human evidence in gynecologic malignancies currently comes from small early-phase, pan-tumor programs and biomarker signals rather than validated GC-specific clinical endpoints (PFS/OS).

Clinical translation remains limited: GC-dedicated interventional trials of lipid-metabolism inhibitors are still scarce, and most evidence for targets such as FASN/ACC/ACLY/SCD1, CD36, and FABP4 is currently preclinical or derived from broader solid-tumor programs rather than validated GC-specific clinical endpoints.

However, to the best of our knowledge, there are no data on drugs (or other procedures listed in Supplementary Table S1) targeting these enzymes, that are being tested in clinical trials in GC patients. These agents may also inhibit lipid metabolism in normal cells of organs affected by cancer or in cells throughout the whole body. This may increase the risk of side effects.

Accordingly, demonstrating a therapeutic window (tumor selectivity vs. systemic metabolic toxicity) and identifying patient subgroups most likely to benefit are critical steps before routine clinical use. These agents may also inhibit lipid metabolism in normal cells of organs affected by cancer or in cells throughout the whole body. This may increase the risk of side effects (Liang and Dai, 2022). This limitation is particularly evident for FAO inhibition: in the ERGO phase II study, the CPT1 inhibitor etomoxir was stopped prematurely due to clinically relevant liver transaminase elevations, highlighting the challenge of systemic CPT1 blockade (Holubarsch et al., 2007). Similarly, perhexiline (a CPT inhibitor used in some cardiovascular settings) requires careful therapeutic monitoring because dose-limiting hepatotoxicity and neuropathy have been reported, underscoring that repurposing FAO inhibitors for oncology would require rigorous safety management (Liang, 2023).

Therefore, the studies investigating the effects of these compounds on both cancer cells and normal cells of the organ are valuable. In addition, the use of targeted therapies–i.e., therapies in which the inhibitor specifically targets cancer cells or cells of a particular organ - appears to be particularly attractive. Thus, further research and clinical trials are needed to evaluate the efficacy and safety of these potential therapeutic approaches and to develop targeted interventions that can affect effectively lipid metabolism in GC.

### 4.2 Clinical/epidemiologic evidence and applicability

It is noteworthy that statins, commonly used drugs to treat hypercholesterolemia, have improved the survival of patients with pancreatic cancer. The potential anticancer effects of statins are associated with their ability to inhibit the mevalonate pathway and the Akt/mTOR pathway, which reduce tumor progression by inducing apoptosis and autophagy (Wang K. H. et al., 2022; Xia et al., 2023). It appears that the effect of statins may also be mediated via a lipid-independent mechanism (Huang et al., 2016). In EC patients, statins are associated with improved overall survival, probably due to their anti-inflammatory and antiproliferative properties (Li et al., 2018; Wang K. H. et al., 2022). In contrast, some studies suggest that statins have no significant effect on the risk of OC. However, in this case, a potential benefit was observed when statins were used in combination with other therapies (Wang Q. et al., 2022; Jiao et al., 2024). Surprisingly, statin treatment was associated with an increased risk of cancer in CC patients, highlighting the need for further research to clarify these associations and optimize treatment strategies (Jiao et al., 2024). Importantly, large prospective observational data (United Kingdom Biobank) reported no clear association between statin use and risk of endometrial or ovarian cancer, but a higher risk of CC among statin users; therefore, any “anticancer” interpretation of statins in GCs should be framed as hypothesis-generating and confounding-prone rather than practice-changing (Jiao et al., 2024).

Across lipid-targeting approaches, separating preclinical “target vulnerability” from clinical “patient benefit” is essential. Future studies should prioritize (i) clear staging of evidence (cell/animal vs. early-phase vs. randomized trials), (ii) standardized metabolic phenotyping (BMI, insulin resistance/diabetes, baseline lipid profile), and (iii) pharmacodynamic confirmation of target engagement in tumor tissue or validated circulating lipid markers.

**FIGURE 2:**
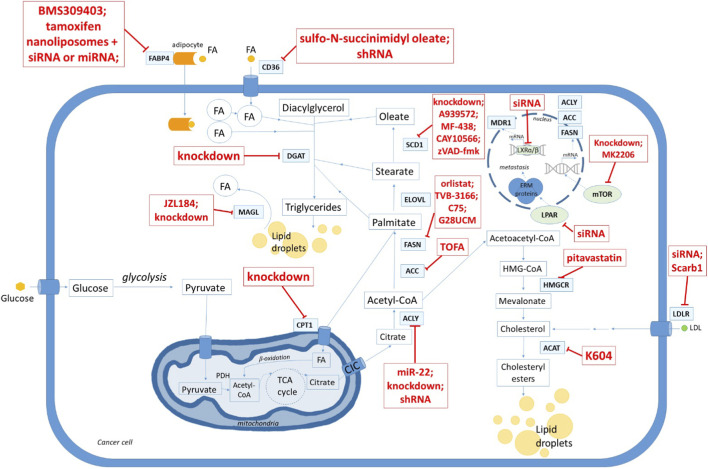
Targeting enzymes and other proteins involved in lipid metabolism for gynecological cancers therapy. The schematic illustrates the main nodes of lipid metabolism that can be targeted in gynecological cancers. Potential interventions include: inhibition of FA uptake (FABP4, CD36), FA synthesis (ACC, ACLY, FASN, SCD1, ELOVL), FA storage (DGAT1, ACAT), and FA oxidation (CPT1). Cholesterol uptake (LDLR) and mevalonate pathway enzymes (HMGCR) are also shown as therapeutic targets. Red boxes indicate representative inhibitors, siRNAs, shRNAs, knockdown approaches, or small molecules currently reported to interfere with these processes in preclinical models. Together, these interventions aim to reduce lipid availability for membrane biosynthesis, signaling, and energy production, thereby impairing cancer cell survival, proliferation, migration, and resistance mechanisms.

The main strategies based on inhibition of lipid metabolism are shown in Figure 2. The current state of research on the inhibition of lipid metabolism in GCs is summarized in more detail in Supplementary Table S1.

## 5 Lipids in the diet - prevention or risk factor for gynecological cancers

Diet is one of many factors that contribute to cancer risk and proper nutrition plays an important role in cancer prevention (Do et al., 2003; Saadatian-Elahi et al., 2004; O’Keefe et al., 2007). Epidemiological studies and randomised clinical trials suggest that dietary n-3 PUFAs are potentially beneficial to human health and may protect against some cancers (Do et al., 2003; Saadatian-Elahi et al., 2004; O’Keefe et al., 2007). In contrast, diet high in SFA and low in PUFA is associated with an increased risk of breast and colon cancer (Do et al., 2003; Saadatian-Elahi et al., 2004; O’Keefe et al., 2007). However, the effect of diet on the risk and prevention of GCs is still an unresolved issue. A major limitation across many observational datasets is residual confounding by adiposity, insulin resistance, and overall energy balance, factors that are tightly correlated with dietary fat intake and strongly linked to GC risk and prognosis.

The results regarding the effect of n-3 PUFA intake on the risk of GCs are also inconclusive. Some studies showed that higher intake of n-3 PUFA was associated with a lower risk of EOC but a higher risk of other ECs (Granados et al., 2007; Hou et al., 2015; Wu et al., 2015). Analysis of data from different populations showed that dietary supplementation with n-3 PUFA inhibits tumor growth and metastatic potential of OC (West et al., 2020). Some data suggest that high levels of n-6 PUFA in the diet increase, while a diet rich in n-3 PUFA reduce the risk of GCs (Granados et al., 2007). Other findings suggest that intake of high n-6 PUFA (GLA) and n-3 PUFA (ALA) from plant sources may be associated with a lower risk of EC (Yammine et al., 2023). It has also been reported that dietary supplementation with n-3 PUFA does not prevent GCs (MacLean et al., 2006; Hoang et al., 2021). Higher intakes of fibre, green leafy vegetables, fish (tuna and salmon) and a favourable PUFA to MUFA ratio, were associated with improved survival after diagnosis of patients with OC (Playdon et al., 2017). Peiqin’s study showed a significant association between higher plasma n-3 PUFA (EPA) levels and EC recurrence (Li et al., 2020). A key interpretive issue is exposure measurement: food-frequency estimates of FA are noisy, while circulating PUFA biomarkers are more objective but may still reflect metabolic state and treatment effects. Thus, inconsistent findings may arise from (a) subtype-specific biology (e.g., endometrioid vs. non-endometrioid EC), (b) timing (pre-diagnosis vs. during/after treatment), and (c) the background n-6/n-3 balance and overall dietary pattern. As for other FAs, no association between high SFA intake and the risk of EC (Hou et al., 2015) or EOC (Wu et al., 2015) was found. In contrast, dietary oleic acid promotes EC (Feng et al., 2020) and CC (Yang et al., 2018) cell growth and metastasis.

Dietary fat may also influence cancer risk indirectly through endocrine pathways, particularly estrogen biology. Women who consumed a high fat diet had higher plasma estrogens concentrations (a risk factors of some GCs) than women who consumed a very low-fat diet (Gorbach and Goldin, 1987). In contrast, women on vegetarian diet had lower plasma and urine concentrations of estrogens than women on omnivorous diets (Adlercreutz et al., 1986; Shultz and Leklem, 2009). These data suggest that high dietary fat increases serum estrogens concentrations, potentially increasing the risk of GCs (Gorbach and Goldin, 1987). Accordingly, a higher intake of fats was related to increased risk of EC (Vecchia et al., 1986), while diet low in fat and high in fruit and vegetables was related to reduced risk of EC (Littman et al., 2001). Moreover, higher intake of cholesterol was associated with a significantly increased risk of EC (Biel et al., 2011; Rodríguez-Miguel et al., 2015). A case-control study with OC patients and control subjects suggested that a diet rich in phytoestrogens, fibre, carotenoids and plant-based foods, may play a protective role in reducing the risk of hormone-related OC (McCann et al., 2003). Furthermore, healthy diet has been shown to support the immune system to help the body fight HPV infection, leading to lower risk of CC and its progression (Nazari et al., 2023).

High-fat diet strategies are heterogeneous: ketogenic diets (KD) contains markedly reduced carbohydrates and increased fat content to induce ketosis (Cohen et al., 2020). In contrast, fasting-mimicking diets (FMD) and other short-term fasting regimens impose time-limited energy (often protein) restriction, which secondarily increases adipose lipolysis and hepatic ketone production; many FMD protocols use cyclic near 5-day periods repeated at intervals (Cohen et al., 2020; Vernieri et al., 2022; Pastora-Sesin et al., 2025). Importantly, although the clinical literature on fasting/FMD is largely pan-cancer, gynecologic cancers are represented in some cohorts: in the Vernieri et al. FMD study, gynecologic cases included ovary (n = 2) and uterus (n = 1), although outcomes were not reported by gynecologic subtype (Vernieri et al., 2022). The clinical literature evaluating fasting/FMD in oncology is methodologically diverse: a 2024 scoping review identified 10 interventional studies (6 randomized controlled trials, 1 controlled clinical trial, 3 single-arm studies; n = 696) across multiple cancer types and fasting schemes, and concluded that evidence is promising but constrained by heterogeneity, small samples, variable adherence, and risk of bias, precluding firm conclusions or routine implementation (Pastora-Sesin et al., 2025). Cohen et al. conducted a randomized controlled trial in women with OC or EC and found that, after a 12-week dietary intervention (lipids assessed at baseline and week 12), a KD did not worsen standard fasting lipid parameters compared with a lower-fat, higher-fibre diet (Cohen et al., 2020). Although clinically reassuring for short-term lipid safety, the brief duration and reliance on standard lipid panels (without apoB/apolipoproteins or particle metrics) mean the trial was not powered enough to determine effects on long-term cardiovascular risk or oncologic outcomes (recurrence, progression-free survival, overall survival) (Cohen et al., 2020). Across broader cancer populations, meta-analyses of controlled trials indicate that KDs tend to produce consistent reductions in fasting glucose and, in several datasets, reductions in triglycerides and IGF-1, while effects on cholesterol fractions are more variable between studies (Amanollahi et al., 2023; Salido-Bueno et al., 2024). Vernieri et al. showed that a cyclic 5-day fasting-mimicking diet was feasible in patients with various cancers and was associated with systemic metabolic remodeling (including reductions in glucose and growth-factor signaling) alongside immune changes; however, these biological effects do not, by themselves, establish cancer-preventive benefit or improved survival (Vernieri et al., 2022). In gynecologic oncology, clinical evidence indicates that KD and FMD are feasible and tolerable but remain insufficiently studied for definitive oncologic conclusions. A randomized controlled trial in OC and EC patients show that KD did not adversely affect lipid profiles while promote loss of overall and visceral fat mass, reduced fasting insulin, and preserved lean mass when compared to a standard low-fat diet (Cohen et al., 2018).

Based on the data published so far, it is reasonable to assume that a higher intake of n-3 PUFAs (EPA, DPA and, DHA) can reduce the risk of GCs, but oleic acid (which is one of the MUFA) may promote the growth and metastasis of EC and CC cells. The effect of n-6 PUFAs on GCs is not yet clear. Many more studies are needed to resolve the issue of the relationship between lipid intake and the risk and prevention of GCs.

## 6 Future challenges

Despite significant progress in understanding the role of lipid metabolism in GCs, several critical challenges remain to be resolved to effectively translate these findings into clinical applications. An understanding of the context-specific role of lipid metabolism in different cancer types and stages is required, especially given the heterogeneity of GCs. This complexity requires precise approaches that target specific subtypes and stages of cancer progression. The identification and validation of biomarkers offers great potential to improve the early diagnosis and prognosis of GCs. These biomarkers may include specific lipid species, enzymes central to lipid metabolism, proteins that mediate lipid transport across cellular and mitochondrial membranes, or signaling molecules that are dysregulated in GCs (Koundouros and Poulogiannis, 2019; Fu et al., 2021). The most promising potential diagnostic biomarkers related to lipid metabolism, that were discussed throughout this review article has been summarized in Table 3 for better readability. Despite the identification of numerous biomarkers related to lipid metabolism in cancer, their clinical applicability remains limited by the lack of standardized validation protocols. To close this gap, large-scale, multicenter studies are needed to determine the sensitivity, specificity and predictive value of these biomarkers, ultimately paving the way for their integration into routine clinical practice. The modulation of lipid metabolism, for example the inhibition of lipogenesis in cancer tissue or a diet with an apropriate lipid composition, could slow the growth of tumors and improve the efficacy of already available therapies such as surgery, chemotherapy, immunotherapy or radiotherapy. Therefore, evaluating the long-term safety, efficacy and potential side effects of lipid therapies is crucial for their successful clinical application. Іdentification of effective dietary and lifestyle interventions for the management of serum lipids levels and metabolism can help in reduction of cancer risk, especially in high-risk individuals (Granados et al., 2007; Fu et al., 2021). These challenges reflect the need for a comprehensive approach to understand and address the role of changes in lipid metabolism in the development of GC. This could be supported by further studies in patients with well-defined female cancers, taking into account the stage, medications, concomitant diseases, and treatment process, as well as studies in cells derived from cancer tumors removed during surgery.

**TABLE 3:** Summary table for lipid-related biomarkers in EC, OC and CC cancers.

Cancer type	Sample matrix	Biomarker(s) tested	Sample size	Validation status	References
Endometrial	Tumor tissue	27-hydroxycholesterol (27-OHC)	n = 126	No	Gibson et al. (2018)
Ovarian	Plasma	LPA (lysophosphatidic acid)	48 OC vs. 48 controls	No	Xu et al. (1998)
	Serum + tumor tissue	Free fatty acid (FFA) signature linked to SCD1 activity	Serum: 41 OC vs. 30 donors; Tissue: 32 tumors vs. 9 normal	Yes (internal discovery/validation split); external validation not reported	Katoh et al. (2023)
EOC	Plasma	Lysophospholipid panel (LPA, LPI, LPC, S1P; 23 species)	117 cases vs. 27 controls (pre-op subset n = 45)	No	Sutphen et al. (2004)
	Plasma + serum	Total plasma LPA vs. CA-125	87 EOC; 74 benign tumors; 50 healthy controls (follow-up subset n = 29)	No	Bese et al. (2010)
Adnexal mass (ovarian/endometrioid enrichment)	Vaginal fluid	LPA (ELISA)	100 women	No	Minis et al. (2019)
Ovarian (adnexal masses)	Plasma	Phospholipid ratios: LPC(20:4)/LPC(18:0); SM(d18:1/24:1)/SM(d18:1/22:0); PC(18:0/20:4)/PC(18:0/18:1)	20 OC; 20 benign; 22 controls	No	Yagi et al. (2020)
EC, OC, CC	Serum/plasma	Standard lipid panel (TG, TC, LDL-C, HDL-C)	Large (varies)	No	See Table 1
EC, OC, CC	Serum/plasma	FA profile	Large (varies)	No	See Table 2

27-OHC, 27-hydroxycholesterol; LPA, lysophosphatidic acid; LPI, lysophosphatidylinositol; LPC, lysophosphatidylcholine; S1P, sphingosine-1-phosphate; FFA, free fatty acids; SCD1, stearoyl-CoA, desaturase-1; EC, endometrial cancer; OC, ovarian cancer; EOC, epithelial ovarian cancer; CC, cervical cancer; TG, triglycerides; TC, total cholesterol; LDL-C, low-density lipoprotein cholesterol; HDL-C, high-density lipoprotein cholesterol.

## 7 Conclusion

The role of abnormalities in lipid metabolism in the development and progression of GCs is increasingly recognized as complex and cancer type specific. The key enzymes of lipogenesis and FAs oxidation are upregulated in GCs, reflecting the metabolic reprogramming required to meet the increased biosynthetic and energetic demands of proliferating cancer cells. The altered expression of FAs transport proteins also underscores the increased FA uptake by GCs cells. Better understanding of lipids disturbances in GCs may provide: a) reliable biomarkers that could be useful for early diagnosis of the disease, and b) inhibitors that affect lipid metabolism, which may improve the effectiveness of current therapeutic strategies for cancer. The data presented in this review also suggest that an adequate diet with appropriate amounts and proportions of lipids, presumably high in n-3 PUFA and low in SFA and MUFA, may have anticancer effects. Apropriate dietary strategies not only contribute to cancer prevention, but could also complement traditional chemotherapy and immunotherapy. However, further studies are needed to a) draw clear conclusions regarding the relationship between abnormalities in lipid metabolism and the development and prognosis of GCs, and b) bridge the gap between basic research and clinical application.
